# Gut microbiome changes with micronutrient supplementation in children with attention–deficit/hyperactivity disorder: the MADDY study

**DOI:** 10.1080/19490976.2025.2463570

**Published:** 2025-02-18

**Authors:** Hayleigh K. Ast, Matthew Hammer, Shiqi Zhang, Alisha Bruton, Irene E. Hatsu, Brenda Leung, Ryan McClure, Priya Srikanth, Yuliya Farris, Lydia Norby-Adams, Lisa M. Robinette, L. Eugene Arnold, Jonathan R. Swann, Jiangjiang Zhu, Lisa Karstens, Jeanette M. Johnstone

**Affiliations:** a Department of Psychiatry, Center for Mental Health Innovation, Oregon Health & Science University, Portland, OR, USA; b Department of Medical Informatics and Clinical Epidemiology, Oregon Health & Science University, Portland, OR, USA; c Department of Human Sciences, The Ohio State University, Columbus, OH, USA; d Faculty of Health Sciences, University of Lethbridge, Lethbridge, AB, Canada; e Pacific Northwest National Laboratory, Richland, WA, USA; f Oregon Health and Science University-Portland State University School of Public Health, Portland, OR, USA; g Helfgott Research Institute, National University of Natural Medicine, Portland, OR, USA; h Department of Psychiatry & Behavioral Health, The Ohio State University, Columbus, OH, USA; i School of Human Development and Health, Faculty of Medicine, University of Southampton, Southampton, UK

**Keywords:** ADHD, children, micronutrients, microbiome

## Abstract

Micronutrients have demonstrated promise in managing inattention and emotional dysregulation in children with attention-deficit/hyperactivity disorder (ADHD). One plausible pathway by which micronutrients improve symptoms is the gut microbiome. This study examines changes in fecal microbial composition and diversity after micronutrient supplementation in children with ADHD (*N* = 44) and highlights potential mechanisms responsible for the behavioral improvement, as determined by blinded clinician-rated global improvement response to micronutrients. Participants represent a sub-group of the Micronutrients for ADHD in Youth (MADDY) study, a double blind randomized controlled trial in which participants received micronutrients or placebo for 8 weeks, followed by an 8-week open extension. Stool samples collected at baseline, week 8, and week 16 were analyzed using 16S rRNA amplicon sequencing targeting the V4 hypervariable region. Pairwise compositional analyses investigated changes in fecal microbial composition between micronutrients versus placebo and responders versus non-responders. A significant change in microbial evenness, as measured by alpha diversity, and beta-diversity, as measured by Bray-Curtis, was observed following micronutrients supplementation. The phylum *Actinobacteriota* decreased in the micronutrients group compared to placebo. Two butyrate-producing bacterial families: *Rikenellaceae* and *Oscillospiraceae*, exhibited a significant increase in change following micronutrients between responders versus non-responders. These findings suggest that micronutrients modulated the composition of the fecal microbiota and identified specific bacterial changes associated with micronutrient responders.

## Introduction

Attention-deficit/hyperactivity disorder (ADHD) impacts 7.6% of children internationally and impairs the ability to focus as well as regulate emotions and behavior in at least two settings, such as home and the classroom.^1^ Nutrient intake, either from diet or supplementation, has been a treatment target in ADHD for more than four decades, with early studies focused on elimination diets,^2,3^ mega dose vitamins,^4^ or single nutrient supplementation.^5–7^ Outcomes ranged from mixed results, to concerning, or limited evidence of benefit.^2–8^ More recently, three randomized controlled trials (RCTs) of broad-spectrum micronutrients or “multinutrients,” containing all known vitamins, essential minerals and antioxidants: two studies in children^9,10^ and one in adults^11^ have shown improvement in ADHD symptoms and emotional dysregulation (specifically, depression in adults).

Vitamins and minerals impact the bacteria residing in the gastrointestinal tract as measured in stool.^12^ These bacterial communities make up part of the gut microbiome. Growing evidence supports the connection between gut microbiota and neurodevelopmental and mental health conditions in children^13^ including ADHD.^14^ In pediatric ADHD, current research is inconclusive in establishing which bacteria are increased or decreased in children with ADHD compared to those without. For example, the phylum *Actinobacteriota* was observed to be more abundant in children with ADHD compared to neurotypical children in two studies,^15,16^ but this finding was not consistent across other studies.^17–19^ In conjunction with conflicting results, comparing microbial findings between studies is complicated due to differences in diet,^20^ location,^21,22^ environmental factors,^23,24^ medication use,^25,26^ and sequencing methodology and bioinformatics.^27^

As the composition, or make-up, of a healthy gut microbiome in pediatric populations is not well defined, this study will focus on bacterial diversity, and the change in abundance of bacterial taxa after micronutrients supplementation. Variations in the abundance of certain microbial features, such as bacterial taxa and alpha diversity, have been associated with specific neurological and psychiatric disorders.^28^ In research on individuals with autism spectrum disorder (ASD), a neurodevelopmental condition which often co-occurs with ADHD,^29^ symptom improvement occurred after interventions like probiotics,^30,31^ fecal microbial transplant,^32^ and dietary changes.^33^ These improvements were attributed to changes in gut microbial composition. Microbial changes in ADHD are also correlated with changes in levels of plasma cytokines,^34^ supporting the hypothesis of bidirectional communication between the gut microbiota and brain in neurodevelopmental conditions like ADHD and ASD. One pilot study on microbiome changes (*N* = 17), derived from a larger randomized controlled trial on micronutrients for children with ADHD,^10^ examined 16S rRNA sequencing in a subsample of males following micronutrient supplementation.^35^ Researchers identified a change in the abundance of specific bacteria in the stools compared to controls, but found no change in alpha diversity, which was interpreted as micronutrients impacting the composition of the gut microbiota.^35^

This preliminary study examined the stool microbiota of a subsample of children with ADHD and emotional dysregulation (*n* = 44) who received micronutrients (*n* = 33) or placebo (*n* = 11) in an 8-week RCT with an open label extension. To our knowledge, it is the largest study to characterize and compare the impact of micronutrient intake on the composition of the stool microbiota in children with ADHD (*n* = 44) who received micronutrients or placebo in an 8-week RCT. This study explores three areas: 1) the impact of micronutrient supplementation compared to placebo exposure on the fecal microbiota after the 8-week RCT, and 2) how microbial changes differed between children whose ADHD-related behavioral symptoms improved following micronutrients intake (responders) and those who did not (non-responders) based on blind, clinician-rated Clinical Global Impression-Improvement, and 3) the microbial changes after receiving placebo for 8-weeks. We hypothesized micronutrients would alter the gut microbiome composition after supplementation, with changes to diversity and abundance of microbial taxa.

## Materials and methods

### Data source

This study examined 16s rRNA amplicon stool sample data collected from a subgroup of children, 6–12 years old, who participated in the Micronutrients for ADHD in Youth (MADDY) study, an eight-week, double-blind, RCT of micronutrients versus placebo.^9^ The 8-week randomized portion of the study was followed by an 8-week open-label portion, where all participants took the micronutrients for eight weeks. Biological samples were collected at baseline, week 8 (end of the RCT), and week 16 (end of the open-label). The 36-ingredient micronutrients contained all known vitamins and essential minerals (see Table S1 for ingredients) while the placebo contained cellulose and riboflavin (necessary to color urine to preserve blind). Stool samples were collected at the three sites; Oregon Health & Science University (OHSU), Ohio State University (OSU), and University of Lethbridge in Alberta, Canada.

At baseline, parents or guardians (hereafter “parents”) completed a comprehensive set of parent-report questionnaires about their child’s health and symptoms from the Child and Adolescent Symptom Inventory-5 (CASI-5) which aligns with the Diagnostic and Statistical Manual of Mental Disorders, 5th Edition (DSM-5) criteria for ADHD. Based on parent report children had to meet criteria for ADHD plus have one symptom from either of the CASI-5 sections, oppositional defiant disorder and disruptive mood dysregulation disorder signifying emotional dysregulation. Sociodemographic data was collected at baseline and body mass index (BMI) was calculated from height and weight measurements at each visit using a stadiometer with an adjustable headpiece and a calibrated digital scale, respectively. The detailed study design and primary outcomes of the MADDY study are provided elsewhere.^9,36^

### Sample

**Figure 1.**
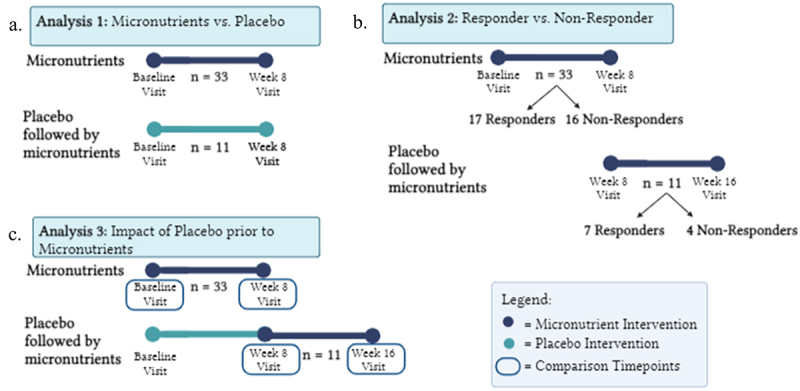
Graphic study design for all analyses of microbiota composition. a) analysis 1 compares children who received micronutrients to those who received placebo from baseline to week 8. b) analysis 2 compares micronutrient responders and non-responders. c) analysis 3 investigates placebo’s potential influence on the fecal microbiome by comparing micronutrients alone versus placebo followed by micronutrients. Created in BioRender. Ast, H. (2025) https://BioRender.com/f93m447.

Stool samples from 50 participants of the 135 in the MADDY study (age range 6–12 years old), were selected for data generation based on their treatment response status, as measured by the dichotomized Clinical Global Impression-Improvement (CGI-I) of “much improved” or “very much improved” for “responder” vs the other 5 ratings, which classify as for “non-responder.” Sample selection and sizes were influenced by sample availability and budgetary constraints. Of the 50 participants, 4 had low DNA yield and 2 had no baseline samples leaving 44 participants for this study. Samples from baseline and week 8 were analyzed for the micronutrient group and baseline, week 8 and week 16 for the placebo group (Figure 1). Leading to a total of 88 paired samples from 44 subjects for micronutrients versus placebo analysis (Figure 1a) and 88 paired samples from the same 44 subjects for responders versus non-responders (Figure 1b).

### Inclusion criteria

Participants met the DSM-5 criteria for ADHD as assessed by parent reports, and at least one symptom of irritability or anger. Participants had to be psychotropic medication-free for at least 2 weeks before the enrollment visit. Antibiotic history was recorded with no restrictions on participation for recent antibiotic use. Participants were permitted to continue previous supplements if they did not contain ingredients in the active treatment; examples of included supplements were fish oil and melatonin. Participants were asked to discontinue probiotics if reported at the initial visit. All participants spoke English. Recruitment started in April 2018 and ended in January 2020 for all three sites.

### Exclusion criteria

Participants were excluded if parents reported a neurological disorder involving central functioning or another major psychiatric condition requiring hospitalization, or other serious medical chronic condition such as inflammatory bowel disease, history of cancer, kidney or liver disease, hyperthyroidism, or diabetes or any known abnormality in mineral metabolism such as Wilson’s disease or hemochromatosis. Additional exclusion for MADDY study can be found in the primary paper.^9^

### Ethics approval

All parents provided consent and children provided assent as approved by Institutional Review Boards at each institution: OHSU (#16870), OSU (2017H0188), the Conjoint Health Research Ethics Board at University of Calgary (#17–0325) for the University of Lethbridge, the US Food and Drug Administration (FDA IND #127832), and Health Canada (Control #207742). The study was prospectively registered with the National Clinical Trials Registry (NCT03252522).

### Outcomes

#### Treatment “responder” or “non-responder”

The primary behavioral outcome was a dichotomized “treatment responder” versus “non-responder” variable based on the blinded clinician-rated CGI-I scale. The CGI-I is a validated standard measure frequently used to evaluate response to an intervention.^37^ The CGI-I ratings, which range from *very much improved* (ranked 1) to *very much worse* (ranked 7), reflect clinical judgment of improvement from baseline to current assessment period. A “treatment responder” was denoted by a CGI-I of 1 or 2 (very much improved or much improved), or a “non-responder,” a CGI-I score of ≥3 (mildly improved to very much worse). The CGI-I was completed at week 8 (end of RCT) and week 16 (end of open label) initially by research staff. The CGI-I was then reviewed at weekly cross-site telephone calls with senior staff using all available information including parent- and child-report questionnaires, qualitative data, and behavior observed during the study visit. All study staff and raters were blind to treatment allocation.

### Study design

This study comprises three pairwise analytical comparisons of change in fecal microbial composition. In the first, samples from two time points: baseline and week 8, were analyzed for the primary outcome comparing micronutrients versus placebo groups change in microbiome composition (Figure 1a). The second analysis compared responders versus non-responders to micronutrients utilizing samples from the first 8 weeks of the study (baseline and week 8) in those who were randomized to micronutrients, initially, and the second 8 weeks (week 8 to week 16) in those who were randomized to placebo first and received micronutrients the second 8 weeks (Figure 1b). A third analysis compared gut microbiome changes in children who received micronutrients in the first 8 weeks of the study versus children who received placebo first followed by micronutrients during the second 8 week period to assess if the preceding placebo altered gut microbiome response (Figure 1c).

### Intervention

The micronutrient capsules contain 36 ingredients including all vitamins and essential minerals, with antioxidants and a proprietary blend of amino acids and herbs. Formula details are included in supplementary material (Table S1). A total of 9 to 12 capsules taken per day, resulted in doses above the Recommended Dietary Allowance (RDA), with seven nutrients above Upper Intake Level (UL) and two nutrients, magnesium and niacin, above the Lowest Adverse Event Level (LOAEL) as determined by the Institute of Medicine Panel on Dietary Antioxidants and Related Compounds. Further details can be found in.^38^ The formula, Daily Essential Nutrients, and placebo were provided by HARDY Nutritionals at no cost. Specific dosages of the nutrients are decided by HARDY nutritionals and influenced by research. The placebo contained cellulose and 0.1 mg of riboflavin per capsule to color the urine to enhance participants’ blinding. Total riboflavin dose in placebo is 0.9–1.2 mg/day, which is above the RDA for children age 4–8 years old (RDA of 0.6 mg/day) and RDA for 9–13 years old (RDA of 0.9 mg/day).^9,39^

### Sample collection

Stool samples were collected using OMNIgene-gut collection kits (www.dnagenotek.com), which provided homogenization and stabilization at the time of collection, at three timepoints: baseline, week 8 (RCT end), and week 16 (open extension end). Parents were provided with kits and complete instructions. Samples were collected at home with parent helping child as needed. Toilet accessories were provided with the kit for a collection free of urine and toilet water. After a bowel movement, an included plastic spatula was used to collect and transfer a small fecal sample into the tube, leveled to remove excess, and capped. Participants were instructed to shake the tube for 30 seconds so the proprietary stabilizing lytic liquid to preserve the sample for 60 days at room temperature. The sample was returned at the next visit (within days of collection) and immediately stored at −80°C until analysis. Samples from Canada and Ohio were shipped on dry ice to OHSU and stored at −80°C until analysis.

#### Microbiota data generation and processing

DNA extraction, 16S rRNA gene sequencing and raw data processing amplifying the V4 hypervariable region were completed at Pacific Northwest National Laboratory (PNNL). DNA was extracted from samples using the Quick-DNA Fecal/Soil Microbe Miniprep Kit (Zymo). Briefly, sequencing was performed on an Illumina MiSeq with 16S using the following primers: 515 V4 region (5'-GTGYCAGCMGCCGCGGTAA-3') and 806 (5'-GGACTACNVGGGTWTCTAAT-3'; 0.2 µM final concentration). Raw fastq files were processed with DADA2 and the Silva SSU database release 138 with the classify-sklearn plugin. Data were rarefied to the lowest number of reads per sample (16,615) for alpha diversity analyses. Alpha diversity measures, which summarize the richness and evenness of the microbial composition within a sample, were calculated on rarefied data using the microbiome R package. We assessed four measures of alpha diversity: 1) Observed taxa summarizing pure richness of the microbial community, 2) Pielou evenness index to summarize the distribution of the microbial community, and the 3) Inverse Simpson and 4) Shannon indices, which are common diversity metrics summarizing the richness and evenness of a community, with the Inverse Simpson index giving more weight to dominant taxa. Beta diversity which summarizes between sample diversity, was evaluated with the Bray Curtis metric calculated on rarefied data using QIME2. The longitudinal beta diversity for each participant between timepoints were calculated using the longitudinal QIIME2 plugin *longitudinal pairwise-distances*.^40^ The initial count data were transformed to relative abundance for taxonomic analyses. Pairwise analyses of individual taxa were used to evaluate change in the composition of the gut microbiome over time highlighting differences in compositional change between the micronutrients and placebo, and between responders versus non-responders. For the purposes of this study compositional change is defined as the change in relative abundance of individual taxa. For each set of analyses, only bacterial families that had a non-zero value in at least 80% of the samples were retained. There was no difference in sequencing depth between groups (*p*-value = 0.59 responders vs non-responders; *p*-value = 0.79 micronutrients vs. placebo).

#### Statistical analysis

Baseline characteristics between the micronutrient and placebo groups, such as age, gender, race and ethnicity, body mass index (BMI) and others were examined, using mean (standard deviation) or number (%). This analysis did not adjust for age, gender, and BMI due to a small sample size increasing the potential of overfitting a model.

The distribution of the data was determined to be normally or nonnormally distributed and a t-test or Mann-Whitney U test (nonparametric alternative to the independent sample t-test) was used, respectively, to compare differences between groups (micronutrient vs placebo groups, responder vs. non-responders, and micronutrient only vs. placebo first groups). All tests were two-tailed and used a non-corrected significance threshold of α = 0.05, as these were exploratory and hypotheses-generating analyses. For transparency, we additionally report the p-values adjusted for multiple comparisons, calculated with the p.adjust function in R using the fdr method. All analyses were performed using R version 4.2.1.

## Results

### Participants and treatment exposure

**Figure 2.**
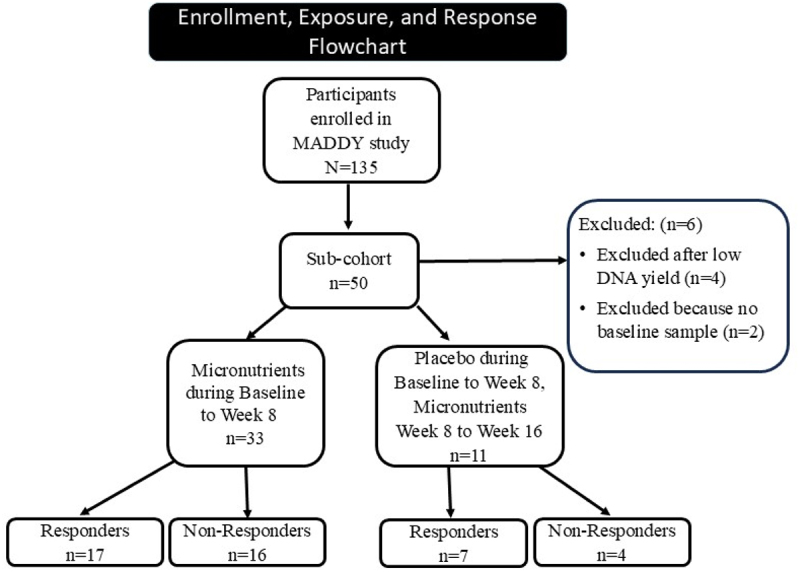
Enrollment, micronutrients exposure, and micronutrients response flowchart categorized and structured based on strengthening the organization and reporting of microbiome studies (STORMS) flowchart recommendations. ^41^ The flow chart does not include analysis of placebo’s impact on microbiome (Figure 1c).

Of the 135 participants in the MADDY study, stool samples from 50 participants were selected to generate data based on participants’ treatment responder status. Of those 50 participants, samples from six participants were excluded: four stool samples sent for data generation returned with low DNA yield, and two had no baseline sample, so could not be included in analysis, yielding data from 44 participants. Thirty-three participants received micronutrients during the first study phase (baseline to week 8; 17 responders and 16 non-responders). The remaining 11 (7 responders and 4 non-responders at week 16) received the placebo during the first study phase (baseline to week 8) and the micronutrients during the second phase (week 8 to week 16) see Figure 2. Two children who received placebo (*n* = 11) were responders at both Week 8 and Week 16.

Baseline characteristics were not significantly different between the treatment groups (Table 1) or in comparing responders versus non-responders except that parental education was higher in responders (*p* = 0.045) (Table S2). The cohort (mean age: 9.6 years) was predominantly male (70.5%), of medium or higher socioeconomic status (61.4% had annual household income >$80,000), and parents who were college educated or higher (70.5%). Previous stimulant use was reported by 41% of participants, meaning they were not medication naive. Potential gut microbiome influences were considered, including the use of antibiotics, probiotics, and laxatives. Three participants were antibiotic-naïve, and the median amount of antibiotics used over the child’s lifetime, based on parent report, was three rounds of antibiotics (Table 1). During the study period, only one participant on placebo required use of one round of oral antibiotics for strep throat before Week 8 stool collection which is noted in limitations. Prior to the study, one participant reported using a probiotic and another reported using a probiotic and a laxative, both participants discontinued use prior to the study.

**Table 1.** Baseline characteristics by treatment group.

	Total	Micronutrient	Placebo
**Characteristic**	**N = 44**	**n = 33**	**n = 11**
Child’s age (years), mean (SD)^a^	9.6 (1.8)	9.7 (1.6)	9.4 (2.2)
Child’s sex, n (%)^b^			
Female	13 (29.5%)	9 (27.3%)	4 (36.4%)
Male	31 (70.5%)	24 (72.7%)	7 (63.6%)
Site, n (%)^b^			
Oregon	20 (45.5%)	16 (48.5%)	4 (36.4%)
Ohio	12 (27.3%)	8 (24.2%)	4 (36.4%)
Canada	12 (27.3%)	9 (27.3%)	3 (27.3%)
Household income, n (%)^b,h^			
≤30K	5 (11.4%)	5 (15.2%)	0 (0.0%)
>30K-≤60K	6 (13.6%)	5 (15.2%)	1 (9.1%)
>60K-≤80K	6 (13.6%)	4 (12.1%)	2 (18.2%)
>80K	27 (61.4%)	19 (57.6%)	8 (72.7%)
Parent Education, n (%)^b^			
High school	6 (13.6%)	5 (15.2%)	1 (9.1%)
Technical/professional college	7 (15.9%)	5 (15.2%)	2 (18.2%)
University or higher	31 (70.5%)	23 (69.7%)	8 (72.7%)
Race or ethnicity, n (%)^c^			
American Indian or Alaskan Native	3 (6.8%)	3 (9.1%)	0 (0.0%)
Asian	3 (6.8%)	3 (9.1%)	0 (0.0%)
Black or African American	4 (9.1%)	2 (6.1%)	2 (18.2%)
White	37 (84.1%)	27 (81.8%)	10 (90.9%)
Other Race^f,g^	2 (4.5%)	2 (6.1%)	0 (0.0%)
Body Mass Index, mean (SD)^a, e^	17.9 (3.9)	17.8 (3.7)	18.3 (4.5)
# of antibiotic rounds since birth, median (IQR)^d^	3.0 (1.0–5.5)	4.0 (1.0–5.0)	2.0 (1.0–6.0)
Treatment Response^b^			
Non-Responder	20 (45.5%)	16 (48.5%)	4 (36.4%)
Responder	24 (54.5%)	17 (51.5%)	7 (63.6%)

^a^two-sample t-test, ^b^chi-square test, ^c^Fisher’s exact test, ^d^Mann-Whitney test.

^e^1 missing BMI.

^f^1 Metis and 1 hispanic among those that said “Other” to Race.

^g^Participants may have selected more than one race to represent multiracial background and results may not add up to 100%.

^h^Reported in United States Dollars and Canadian Dollars in their respective countries.

Acronyms: SD, standard deviation; K, thousand; IQR, Interquartile Range.

There were no significant differences between micronutrient and placebo groups.

### Gut microbiome sequencing

In the dataset, there were 92 families, 277 genera. The top ten most abundant taxa were assessed due to the sparsity of the data and longitudinal study design.

### Analysis 1: Micronutrients (n = 33) vs. placebo (n = 11)

#### 1a: Community diversity

**Figure 3.**
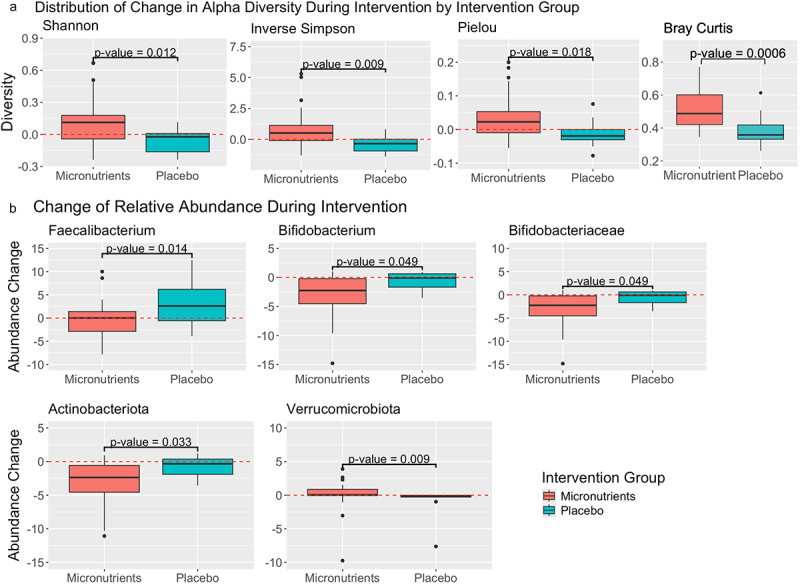
Distribution of change in alpha diversity and abundance of specific taxa baseline to week 8 by micronutrients and placebo. A) the distribution of change in alpha diversity (within sample community diversity) during intervention for each intervention group at the family level. See table S3 for median [IQR] values. The Y-axes represent the change in each respective alpha diversity metric during intervention (value after intervention – value before intervention = change during intervention). B) the distribution of change in relative abundance during intervention for each intervention group, for select taxa that exhibited a significant difference between intervention groups. *Faecalibacterium* and *bifidobacterium* are genera, *bifidobacteraceae* is a family, and A*ctinobacteriota* and *Verrucomicrobiota* are phyla. The Y-axes represents the change in relative abundance during intervention (relative abundance after intervention – relative abundance before intervention = change in relative abundance during intervention). In both figures, the X-axis represents the response group for which each distribution belongs to. Those subjects in the “placebo” intervention group received a placebo during the timeframe of this analysis, whereas subjects in the “micronutrients” intervention group received micronutrients. Zero (indicating no change) is indicated by a red dashed line. P-values here were not corrected for multiple testing and correspond to the significance of the difference in distribution of change in alpha diversity, between intervention groups, as calculated using a Wilcoxon rank-sum test for significance.

At the genus level, the placebo group from baseline to week 8 showed a significant within group decrease in Inverse Simpson alpha diversity. No significant differences in the *change* in alpha diversity were found at the genus level between intervention groups. At the family level, significant increases in Inverse Simpson and Pielou alpha diversity were observed within the micronutrients group, but not placebo. The within-group change in Shannon alpha diversity was not significant in either micronutrient or placebo groups (Table S3). Significant differences in the *change* in Shannon, Inverse Simpson, and Pielou alpha diversity were observed between micronutrient and placebo groups at the family level (Figure 3a and Table S4). Regarding between group beta diversity, we observed a significant increase in the change in beta diversity after micronutrient supplementation compared to placebo (median [IQR] micronutrients 0.49 [0.42, 0.60], placebo 0.36 [0.33,0.2] *p* = 0.0006) (Figure 3a).

#### 1b: Taxa abundance analysis

##### Genus level

Significant increases within groups were seen in the abundance of *Bacteroides* from baseline to week 8 in the micronutrients and placebo groups. Significant decreases within the micronutrients group were observed in the abundance of *Agathobacter*, *Blautia*, *Subdoligranulum*, and *Bifidobacterium*. Even though the difference in the abundance of *Faecalibacterium* was not significant, the abundance increased within the placebo group while remaining unchanged in the micronutrient group (Table 2). Significant differences in the *change* in abundance of *Faecalibacterium* and *Bifidobacterium* were observed between the groups at the genus level (Figure 3b).

**Table 2.** Significant within group changes in bacterial abundance with micronutrients or placebo from baseline to week 8.

Taxonomic Level	Taxa	Intervention Group	Before (mean % (SD)) or (median % [IQR])	After (mean % (SD)) or (median % [IQR])	*p*-value	*p*-adj
Genus	*Bacteroides*	Placebo	7.73* [5.08, 9.56]	11.84* [9.76, 14.92]	0.008	0.080
		Micronutrients	8* [3.73, 11.63]	11.64* [7.75, 17.02]	0.027	0.054
	*Agathobacter*	Micronutrients	3.39* [1.85, 4.33]	1.32* [0.76, 2.60]	0.005	0.013
	*Blautia*	Micronutrients	9.52* [8.22, 10.78]	6.99* [4.31, 8.01]	0.005	0.013
	*Subdoligranulum*	Micronutrients	3.11* [2.53, 4.97]	2.01* [1.40, 3.62]	0.003	0.013
	*Bifidobacterium*	Micronutrients	4.31* [1.37, 7.29]	1.36* [0.41, 2.87]	0.001	0.01
		Micronutrients	10.74 (5.31)	8.95 (4.78)	0.156	0.200
Family	*Bacteroidaceae*	Placebo	7.73* [5.08, 9.56]	11.84* [9.76, 14.92]	0.008	0.080
		Micronutrients	8.00* [3.73, 11.63]	11.64* [7.75, 17.02]	0.008	0.080
	*Bifidobacteriaceae*	Micronutrients	4.31* [1.37, 7.29]	1.36* [0.41, 2.87]	0.001	0.010
	*Lachnospiraceae*	Micronutrients	36.22 (10.18)	31.73 (7.57)	0.047	0.118
	*Rikenellaceae*	Micronutrients	1.26* [0.74, 1.88]	2.06* [1.15, 3.23]	0.017	0.085
Phylum	*Actinobacteriota*	Micronutrients	4.62* [1.63, 7.99]	1.44* [0.43, 3.24]	<0.001	<0.001
	*Firmicutes*	Micronutrients	78.92* [73.36, 83.54]	74.4* [65.30, 78.02]	0.034	0.153
	*Bacteroidota*	Micronutrients	13.65* [10.86, 18.61]	21.14* [15.12, 29.91]	0.004	0.036

Values in the “Before” and “After” columns represent the center of the distribution of relative abundance values for each bacterial taxa in each intervention group, before or after intervention. These values correspond to the mean relative abundance if the value is normally distributed or to the median relative abundance if the value is non-normally distributed (denoted by a * next to the value). P-values denote the significance of the difference between “Before” and “After” values for each bacterial taxa in each intervention group, determined using a Wilcoxon rank-sum test within-group change. P-adj represent p-values with an fdr correction for multiple comparisons.

##### Family level

A significant within group increase in the abundance of *Bacteroidaceae* was observed in both groups. Significant decreases in the abundance of families, *Bifidobacteriaceae* and *Lachnospiraceae*, and a significant increase in the abundance of *Rikenellaceae*, were observed within-group changes only in the micronutrients group (Table 2). A significant difference in the *change* in abundance of *Bifidobacteriaceae* at week 8 was observed between groups at the family level (Figure 3b).

##### Phylum level

**Figure 4.**
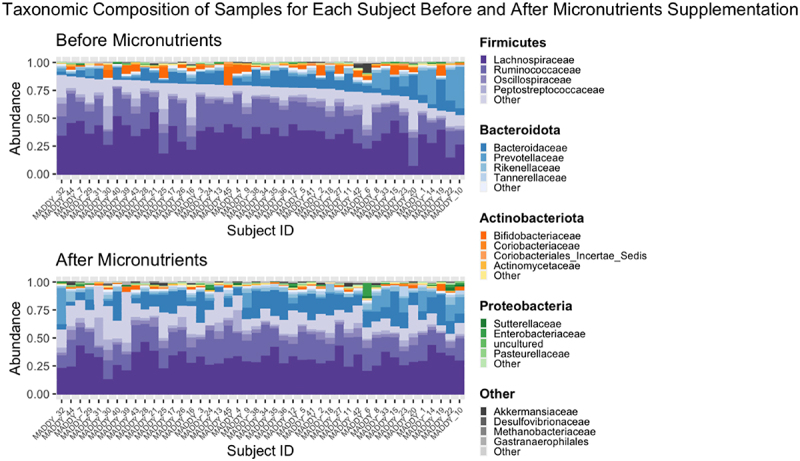
Stacked relative abundance taxa bar plot demonstrating compositional breakdown of each subject’s gut microbiome, before and after micronutrients baseline to week 8. The Y-axis represents the percent abundance of each member of the gut microbial community and totals to 1. The X-axis represents samples, collected before and after micronutrients, for each subject and is ordered by the relative abundance of *Firmicutes* in samples collected before micronutrients, in descending order. Each color represents a distinct microbial phylum, whereas each shade represents a distinct microbial family.

**Figure 5.**
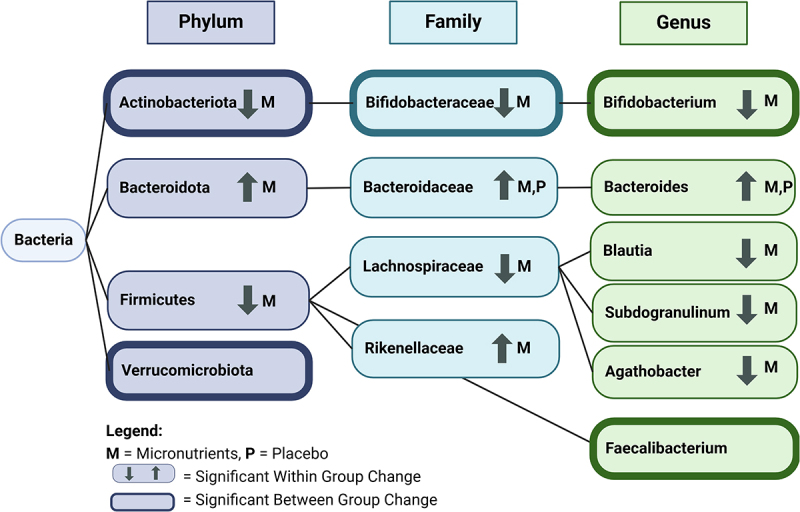
Summary of bacteria significantly changed through 8 weeks of micronutrients or placebo. Arrows indicate significant change within group in taxa abundance and a weighted outline indicates significant between group change in taxa abundance. For example, *Actinobacteriota* has decreased within group change in abundance within the micronutrients group and significant between group change in micronutrients compared to placebo. *Bacteroidaceae* had increased within group change in abundance in both micronutrients and placebo groups. *Verrubomicrobiota* changed significantly between groups. Significance at *p* < 0.05. Created in BioRender. Ast, H. (2025) https://BioRender.com/d23w192

At the phyla level, significant decreases in the abundance of *Actinobacteriota* and *Firmicutes* and a significant increase in the abundance of *Bacteroidota*, were observed within the micronutrients group (Table 2). A significant decrease in the relative abundance of *Actinobacteriota* is depicted in Figure 4, which visualizes the taxonomic composition of all subjects before and after micronutrients. *Actinobacteriota* and *Verrucomicrobiota* exhibited significant differences in the *change* in abundance following intervention between groups (Table S5 , Figure 3b). A summary of significant findings from this analysis are detailed in Figure 5.

### Analysis 2: Responders (n = 24) vs non-responders (n = 20)

#### 2a: Community diversity

At the genus level, no significant within-group differences in alpha diversity were observed in either responders or non-responders. At the family level, significant within-group increases in alpha diversity as measured by the Inverse Simpson were observed in both responder groups (Table 3). Significant within-group increases in Pielou and Shannon alpha diversity measures were observed in non-responders to micronutrients. No significant between-group difference was observed at any taxonomic level following micronutrients between responder groups. A longitudinal beta diversity analysis revealed no statistical difference in the pairwise beta diversity distance between responders and non-responders (median [IQR]:responder 0.49 [0.43, 0.59], non-responder 0.43 [0.041, 0.56], *p* = 0.60).

**Table 3.** Significant within-group changes in alpha diversity following micronutrient treatment by response group.

Taxonomic Level	Diversity Metric	Response	Before Micronutrients (mean (SD)) or (median [IQR])	After Micronutrients (mean (SD)) or (median [IQR])	*p*-value	*p*-adj
Family	Inverse Simpson	Non-Responders	4.36* [3.73, 5.22]	4.81* [4.44, 6.14]	0.030	0.064
		Responders	4.26* [3.74, 5.52]	5.36* [4.62, 6.36]	0.017	0.068
	Pielou	Responders	0.56 (0.06)	0.59 (0.05)	0.035	.084
	Shannon	Responders	1.99 (0.30)	2.13 (0.22)	0.048	0.084

Response groups differentiate between subjects that exhibited a response to micronutrients and non-responders. Values in the “Before” and “After” column represent the center of the distribution of alpha diversity values for each metric, in each response group, before or after micronutrient treatment. These values correspond to the mean alpha diversity value if the value is normally distributed, or to the median alpha diversity value if the value is non-normally distributed (denoted by a * next to the value). P-values denote the significance of the difference between “Before” and “After” values for each metric in each response group within-group change determined using a Wilcoxon rank-sum test. P-adj represent p-values with an fdr correction for multiple comparisons.

#### 2b: Taxa abundance

##### Genus level

The change in abundance of the genera within responder and non-responder groups revealed a significant within-group decrease in the abundance of *Agathobacter* and *Bifidobacterium* (Table 4). In responders a significant within-group decrease in *Blautia* and a significant increase in *Bacteroides* were observed (Table 4). No significant between-group differences in the change in abundance were observed.

**Table 4.** Significant bacterial abundance within-group change following micronutrient treatment by response group.

Taxonomic Level	Taxa	Response	Before Treatment (mean % (SD)) or (median % [IQR])	After (mean % (SD)) or (median % [IQR])	*p*-value	*p*-adj
Genus	*Agathobacter*	Non-Responders	3.69* [1.24, 4.96]	1.08* [0.63, 1.92]	0.017	0.057
		Responders	2.99* [2.29, 4.66]	1.68* [0.90, 3.31]	0.017	0.057
	*Bifidobacterium*	Non-Responders	3.26* [1.61, 7.64]	1.25* [0.13, 2.87]	0.023	0.12
		Responders	2.71* [1.31, 4.81]	1.36* [0.50, 2.01]	0.002	0.020
	*Blautia*	Responders	8.96* [7.22, 9.85]	6.91* [4.59, 8.21]	0.035	0.086
	*Bacteroides*	Responders	10.59* [7.85, 13.87]	15.73* [9.25, 18.35]	0.043	0.086
Family	*Bifidobacteriaceae*	Non-Responders	3.26* [1.61, 7.64]	1.25* [0.13, 2.87]	0.023	0.23
		Responders	2.71* [1.31, 4.81]	1.36* [0.50, 2.01]	0.002	0.020
	*Bacteroidaceae*	Responders	10.59* [7.85, 13.87]	15.73* [9.25, 18.35]	0.043	0.086
	*Rikenellaceae*	Responders	1.35* [0.64, 2.12]	2.86* [1.54, 3.86]	0.009	0.045
	*Oscillospiraceae*	Responders	2.58* [1.45, 4.24]	4.48* [2.73, 5.85]	0.048	0.120
	*Lachnospiraceae*	Non-Responders	38.19 (8.42)	32.73 (8.46)	0.048	0.120
Phylum	*Actinobacteriota*	Non-Responders	3.85* [1.93, 8.27]	1.54* [0.27, 3.19]	0.025	0.250
		Responders	3.01* [1.59, 5.84]	1.43* [0.65, 2.43]	0.003	0.030
	*Bacteroidota*	Responders	18.82 (10.42)	24.83 (9.50)	0.042	0.210

Response groups differentiate between participants that exhibited a clinical response to micronutrients and those who did not. Values in the “Before” and “After” column represent the center of the distribution of relative abundance values for each bacterial taxa in each response group, before or after micronutrients. These values correspond to the mean relative abundance if the value is normally distributed or the median relative abundance if the value is non-normally distributed (denoted by a * next to the value). P-values denote the significance of the difference between “Before” and “After” values for each bacterial taxa in each response group within-group change, determined using a Wilcoxon rank-sum test. P-adj represent p-values with an fdr correction for multiple comparisons.

##### Genus level

**Figure 6.**
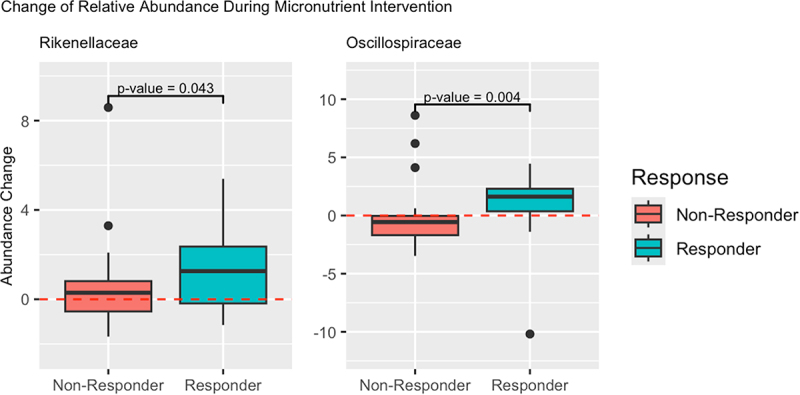
Distribution of between group change in relative abundance following micronutrients by response group. The Y-axis represents the change in relative abundance during micronutrient intervention (change in relative abundance during micronutrient intervention = relative abundance after micronutrients – relative abundance before micronutrients). The X-axis represents the response group. Subjects in the “responder” response group exhibited a positive response to micronutrient intervention, whereas subjects in the “non-responder” response group did not. P-values were not corrected for multiple testing and correspond to the significance of the difference in distribution of change in abundance of the select taxa, between response groups, as calculated using a Wilcoxon rank-sum test for significance.

At the family level, significant within-group decreases in the abundance of *Bifidobacteriaceae* were observed in both responder groups after micronutrients (Table 4). Significant increases in the abundance of *Bacteroidaceae*, *Rikenellaceae*, and *Oscillospiraceae* were observed within the responders group, whereas a significant decrease in the abundance of *Lachnospiraceae* was observed within the non-responders group (Table 4). Significant between-group differences in the *change* in abundance of *Rikenellaceae* and *Oscillospiraceae* were observed following micronutrients (Figure 6). Responders had significantly more *Rikenellaceae* than non-responders (1.26 [IQR: −0.19, 2.36]; 0.29 [IQR: −0.55,0.81], *p* = 0.043). Responders had significantly more *Oscillospiraceae* than non-responders (1.62 [0.36, 2.30]; −0.56 [−1.69,-0.03] *p* = 0.004).

##### Phylum level

**Figure 7.**
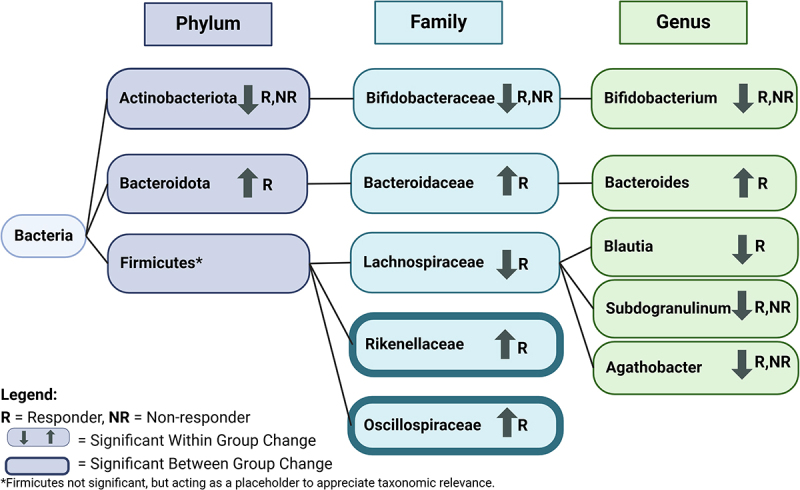
Significant bacterial changes identified in responder vs. non-responder analysis based on taxonomic rank. Arrows indicate significant change within group in taxa abundance and a weighted outline indicates significant between group change in taxa abundance. For example, *Rikenellaceae* had increased within group change in abundance in responders and significant between group change between responders and non-responders, and *Bifidobacterium* had decreased within-group change in abundance in responders and non-responders. Significance indicated at *p* < 0.05. Created in BioRender. Ast, H. (2025) https://BioRender.com/k06g491

At the phyla level, a significant within-group decrease in the abundance of *Actinobacteriota* was observed in both responder groups, and a significant increase in the abundance of *Bacteroidota* was observed in non-responders (Table 4). No significant between-group changes in abundance were observed for any phyla. A summary of significant findings from this analysis is detailed in Figure 7.

### Analysis 3: Impact of placebo prior to micronutrients

**Figure 8.**
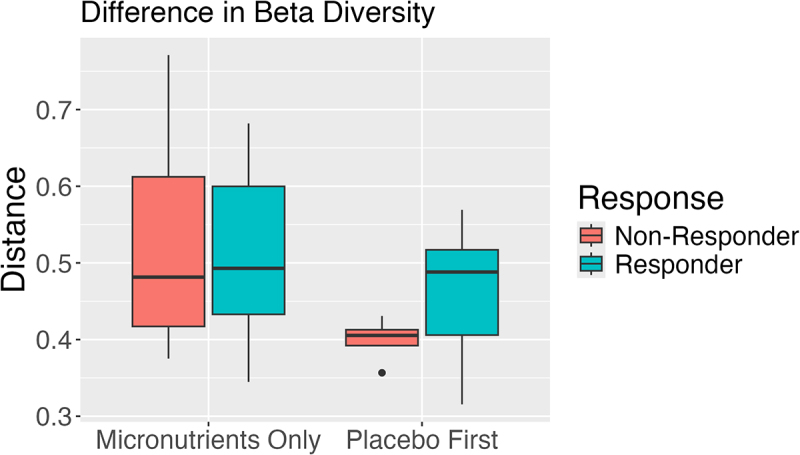
Distribution of change in beta diversity by intervention and response group. Children who received micronutrients during the RCT and responded (*n* = 17) or did not respond (*n* = 16) are compared to children who received placebo first and responded (*n* = 7) or did not respond (*n* = 4) to micronutrients during week 8 to week 16. The y-axis represents the beta-diversity distance between each individuals’ sample before and after micronutrient supplementation. The x-axis represents the intervention group with responders and non-responders depicted by color.

To determine if the placebo affected the gut microbiome, children who received micronutrients for the first 8 weeks were compared to children who received placebo first, followed by micronutrients (week 8 to week 16) (Figure 1c). No significant differences in the change in alpha diversity following micronutrients were observed between groups at any taxonomic level. In terms of beta diversity, we observed a decrease in the pairwise change in beta diversity between individuals who received a placebo prior to micronutrients compared to those who did not (median[IQR] placebo 0.43[0.39,0.49], no placebo 0.49[0.42, 0.60], *p* = 0.057). Interestingly, children who received placebo first and did not respond to micronutrients had a decrease in beta diversity versus responders, although the small sample size should be noted (*n* = 4 non-responder, *n* = 7 responder, Figure 8). A significant between-group difference in the *change* in abundance of *Proteobacteria* following micronutrients was observed between the children who received micronutrients first compared to those who received placebo first followed by micronutrients (Table S6 , Figure S1).

## Discussion

In this study, 8 weeks of micronutrient supplementation intake altered the fecal microbiota of children with ADHD compared to those receiving a placebo. Alterations included a significant change in evenness, as measured by alpha diversity, between sample diversity, as measured by beta diversity, and alterations in the abundance of bacteria that were associated with treatment response. Specifically, metataxonomic analysis identified a reduction in *Actinobacteriota* abundance following micronutrient intake compared to placebo, while an increase was noted in the families, *Oscillospiraceae* and *Rikenellaceae*, in those children with a positive behavioral response to micronutrients.

Variation was observed in alpha diversity after micronutrients versus placebo. Here Inverse Simpson Index and Pielou’s evenness was increased with micronutrient intake but decreased with placebo. This indicates that micronutrients promote diversification of the gut microbiota in children with ADHD. No significant differences were observed in the diversity between micronutrient responders and non-responders, suggesting that while micronutrients lead to a significant increase in alpha diversity compared to placebo, they do not mediate the response to micronutrients. Beta diversity was significantly different in the micronutrients group versus placebo and non-responders. This increase in diversity is different than previous research where no significant difference in beta-diversity was observed after vitamin supplementation over a 4-week period.^12^ The difference in this study includes the longer time-period of 8-weeks and micronutrients, a supplement which includes all essential vitamins and minerals that may augment microbial diversity greater than vitamins alone. Interestingly, the decrease in beta diversity in the non-responders placebo group compared to the responders placebo group in analysis 3 suggests that an increase in diversity may relate to response status. However, the small sample size and lack of significance in analysis 2 (responder versus non-responders) prevents us from confidently concluding this.

Micronutrient supplementation resulted in a significant decrease in the abundance of the phylum *Actinobacteriota* compared to those who received placebo. This is consistent with a previous study measuring gut microbial changes in children with ADHD following micronutrient intake.^35^ As this phylum has been shown to be elevated in children with ADHD,^15,16^ a micronutrient associated reduction could be considered beneficial. However, the degree of change in the *Actinobacteriota* was not related to response, therefore reduction in *Actinobacteriota* was not linked with symptom improvement in this study.

*Oscillospiraceae*, a family under the phylum Firmicutes, significantly increased in micronutrient responders. A significant increase in relative abundance of this family has previously been observed in healthy children and those with inattentive versus combined presentations of ADHD.^42^ No other studies have identified a positive relationship between ADHD and *Oscillopiraceae*. However, *Oscillospiraceae* is closely related to *Ruminococcaceae.*^43^ Mixed results in the literature are observed for abundance of *Ruminococcaceae* in ADHD. Two studies, one RCT and one cross-sectional analysis, demonstrated elevation in the genus *Ruminococcus* in children with ADHD^16,44^ and one cross sectional study demonstrated a decreased abundance in children with ADHD.^20^ Results from this investigation did not show any significant differences in the change in abundance of any genera between responders and non-responders. However, this may be an artifact of the limited taxonomic resolution associated with 16S rRNA amplicon sequencing, as many bacteria in this investigation were assigned taxonomy at the family level, but not the genus level. This lack of taxonomic resolution likely contributed to discrepancies between genus level and family level analyses, which could be the source of inconsistencies between this investigation and previous studies of similar nature.

*Rikenellaceae*, the other bacterial family under the *Firmicutes* phylum identified as playing a potential role in the micronutrient responders, was elevated in children with ADHD^28^ and ASD.^45^ No clear conclusions can be drawn from these limited previous findings. Yet, in childhood obesity, *Rikenellaceae* has been identified as a biological marker to distinguish between obesity and controls.^46^ As analysis was not completed with obesity due to a small sample size we cannot draw conclusions, but future directions may include considering weight as a moderator impacting our current findings.

Interestingly, *Rikenellaceae* and *Oscillospiraceae* families are both butyrate producers.^47^ Butyrate, a short chain fatty acid, can modulate immunity, control inflammation, and influence gene expression.^48,49^ Within the family *Ruminococcaeae*, which is closely related to *Oscillospiraceae*, *Ruminococcus flaveciens* can degrade cellulose from plant material creating short chain fatty acid metabolites like butyrate.^19^ Both the micronutrients and placebo supplements contained cellulose, which may have contributed to the increase in butyrate-producing bacteria like *Ruminococcaeae*. Increases in these butyrate-producing bacteria may be clinically applicable as knowledge of butyrate and the microbiome expands. Most recently, short-chain fatty acids were observed to be reduced in children with ADHD compared to healthy controls,^50^ suggesting that an increase in short-chain fatty acids may influence disorder symptoms. Further these bacteria could be relevant in a common clinical triad: ADHD, atopic disorders, and asthma/allergies, which are all associated with an immune pathway called the T helper 2 (Th2) pathway.^51^ The Th2 pathway relates to the significant bacteria found in this study, as *Oscillospiraceae* is reduced in inflammatory conditions, such as asthma, compared to healthy controls^52^ and *Rikenellaceae* is positively related to allergies.^53^ Increasing short chain fatty acids early in life can address inflammation and reduce risk of Th2 immune reactions like atopy, asthma, and allergies.^49^ Future studies may observe changes in inflammatory markers in correlation with gut bacteria.

In addition to reducing inflammatory cytokines,^54^ short chain fatty acids can impact neurotransmitters such as dopamine, norepinephrine, and serotonin by increasing the rate-limiting enzymes, tyrosine hydroxylase and tryptophan hydroxylase, which produce these neurotransmitters.^55,56^ As changes in neurotransmitter regulation like dopamine are theorized to be correlated with ADHD symptoms, the connection between short chain fatty acids and neurotransmitters is a potential future research direction. Further, mental health disorders such as major depressive disorder, bipolar disorder, psychosis, and schizophrenia are associated with a reduction in butyrate-producing anti-inflammatory microbiota.^57^ An increase in butyrate-producing bacteria like *Rikenellaceae* and *Oscillospiraceae* may have contributed to global symptom improvement observed in micronutrient responders compared to non-responders in our study population.

The placebo composed of cellulose and riboflavin may have primed microbial communities prior to micronutrients in the second phase of the study (Week 8 to Week 16). Riboflavin can influence bacterial changes at doses greater than 30 mg in adults.^58^ The placebo contained 0.9–1.2 mg/day of riboflavin, which may be insufficient to impact change. The community diversity analysis completed on participants who received micronutrients revealed an increased abundance of *Proteobacteria* in participants who received placebo first. *Proteobacteria* can be influenced by riboflavin as demonstrated in adults.^12^ The change in abundance of *Proteobacteria* may have influenced the composition of the gut microbiome of participants who received placebo first. A larger sample size is indicated to confirm results and the impact of riboflavin in the placebo group.

After analyzing all samples from the original RCT (*N* = 135), future investigation for treatment implementation may include designing a probiotic formula with bacteria that increased in relative abundance in micronutrient responders but not in placebo or non-responders. It would be advantageous to compare this novel probiotic to an over-the-counter probiotic and a placebo to appreciate the differences in a specialized formula versus a generic formula. A failed trial would result in an equally effective over-the-counter probiotic while a successful trial would result in a specialized probiotic having greater effect than the over-the-counter probiotic. An alternative to a probiotic formula would be a specialized dietary intervention which targets bacteria in this study that increased in abundance in micronutrient responders, *Oscillaspiraceae* and *Rikenellaceae*. A successful trial would result in positive symptom response to dietary adjustments and altered fecal microbiome to prefer the specified bacteria.

In summary, micronutrients altered the composition of the gut microbiome compared to placebo and the composition of micronutrient responders vs non-responders in children with ADHD. The alpha diversity as measured by Pielou’s evenness and Inverse Simpson was significantly different between children who received micronutrients versus placebo. *Actinobacteriota* significantly decreased in abundance in children who took micronutrients compared to placebo, reaffirming previous studies in children with ADHD and presenting *Actinobacteriota* as a future investigatory marker for the ADHD microbiome. Both butyrate producers, *Oscillospiraceae* and *Rikenellaceae* were significantly increased in abundance in children who responded to micronutrients versus non-responders, which introduces the possibility of immune changes influencing response to micronutrients.

## Limitations

Limitations to this study include those specific to the original study and those common to compositional analyses of the gut microbiome. One study-specific limitation, common to many microbiome studies, is the homogeneity of the subject population. The study sample was predominantly of White race, high socioeconomic status, and high parental education, which limits generalizability of results as gut microbiome composition may vary significantly among different sub-populations. The findings from this study are from medication free or naive children living with ADHD, as such they are not generalizable to children with ADHD taking stimulants. Two participants were on probiotics at baseline potentially influencing the gut microbiome. One participant who received placebo first needed an antibiotic at week 8 which may have influenced their fecal sample. Not accounting for dietary intake is a potential limitation; however, since this study is looking at intra-individual changes attributed to the 8-weeks of micronutrients/placebo, and dietary intake itself was not subject of the intervention, the effect of dietary intake is assumed to be minimal. Future studies are planned to further explore the relationship between diet and microbiome to test this assumption. No *a priori* statistical power was determined due to the small sample size, which was constrained by the available funding.

## Supplementary Material

Supplemental Material

## Data Availability

Data presented in this manuscript will be made available upon request to corresponding authors directly. Data includes: a metadata table with child age in years, child sex, broad geographic location (Oregon or Ohio), response, intervention timepoint, and intervention group, an amplicon sequence variant (ASV) table with counts of each ASV per sample, a taxonomy table with bacterial taxonomy of each ASV.
