# Supplementary material for: Gut microbiome changes with micronutrient supplementation in children with attention–deficit/hyperactivity disorder: the MADDY study
Source: Gut Microbes. 2025 Feb 18;17(1):2463570. doi: 10.1080/19490976.2025.2463570 (PMC11845018; doi:10.1080/19490976.2025.2463570)
Supplement: Supplemental Material [file KGMI_A_2463570_SM8637.zip › Supplementary_Materials_Ast clean.docx]

| **Table 1. Daily Essential Nutrients by Hardy Nutritionals Ingredients and the Institute of Medicine’s Dietary Reference Intakes** | | | | | | | | | |
| --- | --- | --- | --- | --- | --- | --- | --- | --- | --- |
| **Ingredient** | **Unit** | **1 Caps** | **6 Caps** | **9 Caps** | **12 Caps** | **UL**  **4-8 y/o** | **UL**  **9-13 y/o** | **RDA^#^ or AI^+^** | **LOAEL^Ө^** |
| Vitamin A (as retinyl palmitate) | mg^a^ | 0.14 | 0.84 | 1.3* | 1.73* | 0.9 | 1.7 | 6 | 10 |
| Vitamin C (as ascorbic acid) | mg | 50 | 300 | 450 | 600 | 650 | 1200 | 45 | 3000 |
| Vitamin D (as cholecalciferol) | IU | 250 | 1500 | 2250 | 3000 | 3000 | 4000 | 600 | 3800 |
| Vitamin E (as d-alpha tocopheryl succinate) | IU | 30 | 180 | 270 | 360 | NE | NE | 16.5 | 750 |
| Vitamin K (as 75% phylloquinone, 25% menaquinone-7) | mcg | 10 | 60 | 90 | 120 | NE | NE | 60 | NE |
| Vitamin B1 (as thiamin mononitrate) | mg | 5 | 30 | 45 | 60 | NE | NE | 0.9 | NE |
| Vitamin B2 (riboflavin) | mg | 1.5 | 9 | 13.5 | 18 | NE | NE | 0.9 | NE |
| Vitamin B3 (as niacinamide)^c,d^ | mg | 7.5 | 45 | 68* | 90***** | 15 | 20 | 12 | 50 |
| Vitamin B6 (as pyridoxine hydrochloride)^e^ | mg | 5.8 | 34.8 | 53* | 70* | 40 | 60 | 1.0 | 500 |
| Folate - B9 (as calcium L-5 methyltetrahydrofolate)^f^ | mcg | 66.6 | 399.6 | 600* | 800* | 400 | 600 | 300 | 5000 |
| Vitamin B12 (as 75% adenosylcobalamin, 25% methylcobalamin) | mcg | 75 | 450 | 675 | 900 | NE | NE | 1.8 | NE |
| Biotin – Vitamin H | mcg | 90 | 540 | 810 | 1080 | NE | NE | 20 | NE |
| Pantothenic acid (as d-calcium pantothenate) | mg | 2.5 | 15 | 22.5 | 30 | NE | NE | 4 | NE |
| Calcium (as NutraTek™ chelation complex) | mg | 110 | 660 | 990 | 1320 | 2500 | 3000 | 1300 | 4000 |
| Iron (as NutraTek™ chelation complex ) | mg | 1.15 | 6.9 | 10.4 | 13.8 | 40 | 40 | 8 | 70 |
| Phosphorus (as NutraTek™ chelation complex ) | mg | 70 | 420 | 630 | 840 | 3000 | 4000 | 1250 | 10200 |
| Iodine (as NutraTek™ chelation complex ) | mcg | 17 | 102 | 153 | 204 | 300 | 600 | 120 | 1700 |
| Magnesium (as NutraTek™ chelation complex )^c,g^ | mg | 50 | 300 | 450 | 600***** | 110 | 350 | 240 | 360 |
| Zinc (as NutraTek™ chelation complex )^h^ | mg | 4 | 24 | 36* | 48* | 12 | 23 | 8 | 60 |
| Selenium (as NutraTek™ chelation complex )^i^ | mcg | 17 | 102 | 153* | 204 | 150 | 280 | 40 | 913 |
| Copper (as NutraTek™ chelation complex )^j,k^ | mg | 0.6 | 3.6 | 5.4* | 7.2 | 3 | 5 | 0.7 | 10 |
| Manganese (as NutraTek™ chelation complex )^l^ | mg | 0.8 | 4.8 | 7.2* | 9.6* | 3 | 6 | 1.9 | 15 |
| Chromium (as NutraTek™ chelation complex ) | mcg | 52 | 312 | 468 | 624 | NE | NE | 25 | NE |
| Molybdenum (as NutraTek™ chelation complex ) | mcg | 12 | 72 | 108 | 144 | 600 | 1100 | 34 | 1500 |
| Potassium (as NutraTek™ chelation complex ) | mg | 20 | 120 | 180 | 240 | NE | NE | 4500 | NE |
| ^Ө^Lowest Observed Adverse Effects Level; ^#^Recommended Daily Allowance; ^+^Adequate Intake; *****Above UL – see guidelines below; NE None Established. | | | | | | | | | |
| Footnotes: Tolerable Upper Intake Levels (UL) and Lowest Observed Adverse Effects (LOAEL) of Vitamins and Minerals Dosed Above UL for Children 4 to 13 years old and Safety rationale​^1^​  ^a^Dosages for Vitamin A are calculated in milligrams of Retinol Activity Equivalents.  ^b^There are limited case reports of hypervitaminosis (symptoms related to cerebrospinal fluid pressure: headache, vertigo, double vision) above 6mg/d in young children; the risk of exceeding UL appears to be small. The IOM acknowledges vitamin A intake above UL can be appropriate in controlled clinical trials.^2^  ^c^Magnesium and niacin doses were above the LOAEL.  ^d^The 2 forms of niacin are grouped together (nicotinic acid and niacinamide [also called nicotinamide]) and UL set low to prevent skin flushing from nicotinic acid. No reports of skin flushing to date with this formula, which contains niacinamide. The IOM acknowledges that the 2 forms are clearly different.^3^  ^e^Neurological issues were reported in 1 adult study at a dose of 500 mg/d.^3^ No cases of toxicity have been found with B6 administration in children.  ^f^The UL was set to prevent masking a B12 deficiency; the formula contains B12, so the risk of masking B12 deficiency is low.^3^  ^g^UL is set because of concerns regarding diarrhea. A 2019 meta-analysis found that children with ADHD had lower Mg levels than those without.^5^  ^h^The primary safety concern is that higher levels may result in an imbalance with copper. As the formula also contains copper, this concern is reduced.^2^ The formula includes zinc and copper in an accepted ratio (range, 5–10:1).^2^  ^i^“The most frequently reported features of selenosis (chronic toxicity) are hair and nail brittleness and loss…. Intake above the UL may be appropriate for investigation within well controlled clinical trials … the UL is not meant to apply to individuals who are receiving selenium under medical supervision.”^5^  ^j^Copper has a NOAEL for adults; no LOAEL reported.  ^k^Copper is above the UL, but below the No-Observed-Adverse Effect-Level (NOAEL) for adults of 10 mg.^2^ The formula also contains zinc, which competes with copper for absorption.  ^l^The LOAEL was not based on actual clinical adverse effects, but on “increases in serum manganese concentrations after 25 days of supplementation.” There are no reports of manganese toxicity in children and adolescents.^6^ | | | | | | | | | |

**References**:

1. Johnstone JM, Arnold LE, Villagomez A, et al. Dr. Johnstone et al. Reply to Dr. Elmrayed. *J Am Acad Child Adolesc Psychiatry*. Published online 2023.
2. Institute of Medicine. Dietary Reference Intakes for Vitamin A, Vitamin K, Arsenic, Boron, Chromium, Copper, Iodine, Iron, Manganese, Molybdenum, Nickel, Silicon, Vanadium, and Zinc. Washington, DC: National Academies Press; 2001.
3. Institute of Medicine, National Academy of Sciences. Dietary Reference Intakes for Thiamin, Riboflavin, Niacin, Vitamin B6, Folate, Vitamin B12, Pantothenic Acid, Biotin, and Choline. National Academy Press; 1998.
4. Huang YH, Zeng BY, Li DJ, et al. Significantly lower serum and hair magnesium levels in children with attention deficit hyperactivity disorder than controls: a systematic review and meta-analysis. Prog Neuro-psychopharmacology Biol Psychiatry. 2019;90:134–141. 10.1016/j.pnpbp.2018.11.012
5. Institute of Medicine, National Academy of Sciences. Dietary Reference Intakes for Vitamin C, Vitamin E, Selenium, and Carotenoids. National Academy Press; 2000.
6. Institute of Medicine (US). Panel on Micronutrients. Dietary reference intakes for vitamin A, vitamin K, arsenic, boron, chromium, copper, iodine, iron, manganese, molybdenum, nickel, silicon, vanadium, and zinc. Washington, DC: National Academies Press; 2002.

**Table S2.** Baseline characteristics by treatment responder status

|  | **Total** | **Responder** |  | **Non-Responder** |
| --- | --- | --- | --- | --- |
|  | **N=44** | **N=24** |  | **N=20** |
| Child's age (years) | 9.6 (1.8) | 9.5 (2.0) |  | 9.8 (1.4) |
| Child’s sex |  |  |  |  |
| Female | 13 (29.5%) | 7 (29.2%) |  | 6 (30.0%) |
| Male | 31 (70.5%) | 17 (70.8%) |  | 14 (70.0%) |
| Site |  |  |  |  |
| OHSU | 20 (45.5%) | 10 (41.7%) |  | 10 (50.0%) |
| OSU | 12 (27.3%) | 7 (29.2%) |  | 5 (25.0%) |
| UofL | 12 (27.3%) | 7 (29.2%) |  | 5 (25.0%) |
| Household income |  |  |  |  |
| <=30K | 5 (11.4%) | 2 (8.3%) |  | 3 (15.0%) |
| >30K-<=60K | 6 (13.6%) | 4 (16.7%) |  | 2 (10.0%) |
| >60K-<=80K | 6 (13.6%) | 2 (8.3%) |  | 4 (20.0%) |
| >80K | 27 (61.4%) | 16 (66.7%) |  | 11 (55.0%) |
| Parent Education |  |  |  |  |
| High school | 6 (13.6%) | 1 (4.2%) |  | 5 (25.0%) |
| Technical/professional college | 7 (15.9%) | 6 (25.0%) |  | 1 (5.0%) |
| University or higher | 31 (70.5%) | 17 (70.8%) |  | 14 (70.0%) |
| American Indian or Alaskan Native | 3 (6.8%) | 1 (4.2%) |  | 2 (10.0%) |
| Asian | 3 (6.8%) | 3 (12.5%) |  | 0 (0.0%) |
| Black or African American | 4 (9.1%) | 2 (8.3%) |  | 2 (10.0%) |
| Native Hawaiian or Pacific Islander | 0 (0.0%) | 0 (0.0%) |  | 0 (0.0%) |
| White | 37 (84.1%) | 21 (87.5%) |  | 16 (80.0%) |
| Other | 2 (4.5%) | 0 (0.0%) |  | 2 (10.0%) |
| Body Mass Index | 17.9 (3.9) | 17.6 (3.1) |  | 18.3 (4.7) |
| Antibiotics since birth | 3.0 (1.0-5.5) | 2.5 (1.0-5.0) |  | 4.0 (2.0-10.0) |

***Data are presented as mean (SD) for continuous measures and n (%) for categorical measures.***
 ***No significant differences between responders and non-responders except non-responder parents had a higher proportion of those with a high school education (25%) compared to responder parents (4%) (p=0.05)***

**Table S3. Significant *Within* Group Alpha Diversity Change Following Micronutrients or Placebo: Baseline to Week 8**

| Taxonomic Level | Diversity Metric | Intervention Group | Before  (mean (SD))  (median [IQR]) | After  (mean (SD))  (median [IQR]) | p-value | p-adj |
| --- | --- | --- | --- | --- | --- | --- |
| Family | Inverse Simpson* | Micronutrients | 4.51^+^ [3.68, 5.58] | 5.21^+^ [4.50, 6.39] | 0.023 | 0.075 |
|  | Pielou* | Micronutrients | 0.56 (0.06) | 0.59 (0.06) | 0.037 | 0.074 |
|  | Shannon | Micronutrients | 2.01 (0.29) | 2.14 (0.23) | 0.056 | 0.074 |

Values in the “Before” and “After” columns represent the center of the distribution of alpha diversity values for each metric, in each intervention group, before or after intervention. These values correspond to the mean alpha diversity value for subjects in the intervention group, if the value is normally distributed for subjects in the intervention group. However, these values correspond to the median alpha diversity value for the intervention group, if the value is non-normally distributed for subjects in the intervention group (denoted by a ”+“ next to the value). P-values denote the significance of the difference between “Before” and “After” values for each intervention group, for each metric within-group change, as determined using a Wilcoxon rank-sum test. P-adj represents p-values with an **FDR** correction for multiple comparisons.

**Table S4. Significant Alpha Diversity Change *Between* Group Differences Following Micronutrients and Placebo: Baseline to Week 8**

| Taxonomic Level | Diversity Metric | Micronutrients  (median change [IQR]) | Placebo  (median change [IQR]) | p-Value |
| --- | --- | --- | --- | --- |
| Family | Shannon | 0.1 [-0.03, 0.18] | -0.03 [-0.20, 0.00] | 0.012 |
|  | Inverse Simpson | 0.44 [-0.97, 0.01] | -0.33 [-0.18, 1.15] | 0.009 |
|  | Pielou | 0.02 [-0.04, 0.00] | -0.02 [-0.01, 0.05] | 0.018 |

Values in the “Micronutrients” and “Placebo” columns represent the median alpha diversity change value for each metric, in subjects that received micronutrients or in subjects that received the placebo, respectively, since these values are non-normally distributed in each intervention group. P-values denote the significance of the difference in change values between subjects that belong to the “Micronutrients” group and the “Placebo” group for each metric, as determined using a Wilcoxon rank-sum test.

**Table S5. Significant Bacterial Taxa Abundance *Between* Group Change Differences Following Micronutrients and Placebo**

| Taxonomic Level | Taxa | Micronutrients  (mean % change (SD))  (median % change [IQR]) | Placebo  (mean % change (SD))  (median % change [IQR]) | p-value |
| --- | --- | --- | --- | --- |
| Genus | *Faecalibacterium* | -1.79 (5.47) | 2.97 (4.71) | 0.014 |
|  | *Bifidobacterium* | -2.25* [-4.52, -0.22] | -0.09* [-1.68, 0.63] | 0.049 |
| Family | *Bifidobacteriaceae* | -2.25* [-4.52, -0.22] | -0.09* [-1.68, 0.63] | 0.049 |
| Phylum | *Actinobacteriota* | -2.44* [-4.57, -0.65] | -0.33* [-1.90, 0.37] | 0.033 |
|  | Verrucomicrobiota | 0.05* [-0.05, 0.87] | -0.1* [-0.29, -0.03] | 0.009 |

Values in the “Micronutrients” and “Placebo” columns represent the center of the distribution of relative abundance change values for each bacterial taxa in subjects that received micronutrients or in subjects that received the placebo, respectively. These values correspond to the mean relative abundance change value for participants in the intervention group, if the value is normally distributed for participants in the intervention group. However, these values correspond to the median relative abundance change value for the intervention group, if the value is non-normally distributed for participants in the intervention group (denoted by a * next to the value). P-values denote the significance of the difference between “Micronutrients” and “Placebo” values for each bacterial taxa, determined using a Wilcoxon rank-sum test.

**Table S6. Significant Bacterial Taxa Abundance *Within* Group Change Following Micronutrients Only for Analysis 3**

| Taxonomic Level | Taxa | Intervention Group | Before  (mean % (SD))  Median % [IQR]) | After  (mean % (SD))  Median % [IQR]) | P-Value |
| --- | --- | --- | --- | --- | --- |
| Genus | *Agathobacter* | Placebo First | 3.9* [2.13, 6.84] | 1.33* [0.95, 2.75] | 0.039 |
|  |  | Micronutrients Only | 3.39* [1.85, 4.33] | 1.32* [0.76, 2.60] | 0.005 |
|  | *Bifidobacterium* | Micronutrients Only | 4.31* [1.37, 7.29] | 1.36* [0.41, 2.87] | 0.001 |
|  | *Blautia* | Micronutrients Only | 9.52* [8.22, 10.78] | 6.99* [4.31, 8.01] | 0.005 |
|  | *Subdoligranulum* | Micronutrients Only | 3.11* [2.53, 4.97] | 2.01* [1.40, 3.62] | 0.003 |
|  | *Bacteroides* | Micronutrients Only | 8* [3.73, 11.63] | 11.64* [7.75, 17.02] | 0.027 |
| Family | *Bacteroidaceae* | Micronutrients Only | 8* [3.73, 11.63] | 11.64* [7.75, 17.02] | 0.027 |
|  | *Rikenellaceae* | Micronutrients Only | 1.26* [0.74, 1.88] | 2.06* [1.15, 3.23] | 0.017 |
|  | *Bifidobacteriaceae* | Micronutrients Only | 4.31* [1.37, 7.29] | 1.36* [0.41, 2.87] | 0.001 |
|  | *Lachnospiraceae* | Micronutrients Only | 36.22 (10.18) | 31.73 (7.57) | 0.047 |
| Phylum | *Proteobacteria* | Placebo First | 1.24* [0.84, 1.58] | 1.79* [1.29, 4.11] | 0.053 |
|  | *Actinobacteriota* | Micronutrients Only | 4.62* [1.63, 7.99] | 1.44* [0.43, 3.24] | <0.001 |
|  | *Bacteroidota* | Micronutrients Only | 15.21 (8.62) | 21.61 (10.22) | 0.008 |

Intervention groups differentiate between subjects that received the placebo prior to receiving micronutrients (Week 8 to week 16) and those who received the micronutrients only (Baseline to Week 8). Values in the “Before” and “After” columns represent the center of the distribution of relative abundance values for each bacterial taxa, in each intervention group, before or after micronutrients. These values correspond to the mean relative abundance value for subjects in the intervention group, if the value is normally distributed for subjects in the intervention group (denoted by a * next to the value). However, these values correspond to the median relative abundance value for the intervention group, if the value is non-normally distributed for participants in the intervention group. P-values here denote the significance of the difference between “Micronutrients Only” and “Placebo First” values for each bacterial taxa, determined using a Wilcoxon rank-sum test.


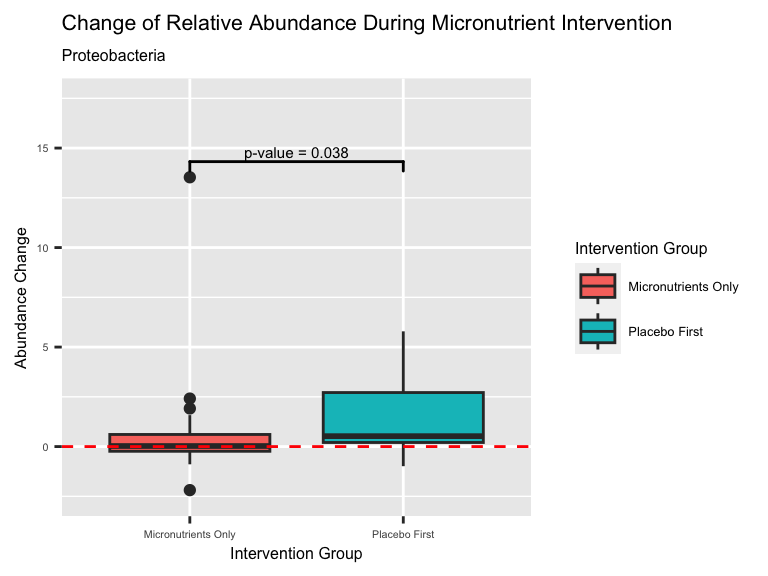


**Figure S1. Distribution of Change in Relative Abundance During Micronutrient Intervention by Intervention Group.** The Y-axis represents the change in relative abundance of *Proteobacteria* during micronutrient intervention (Relative Abundance After Micronutrients – Relative Abundance Before Micronutrients = Change in Relative Abundance During Micronutrient Intervention). The X-axis represents the intervention group for which each distribution belongs to. Those subjects in the “Placebo First” intervention group received a placebo prior to micronutrient intervention, whereas subjects in the “Micronutrients Only” intervention group received only micronutrients. P-value was not corrected for multiple testing and correspond to the significance of the difference in distribution of change in abundance of the select taxa, between intervention groups, as calculated using a Wilcoxon rank-sum test for significance.
